# Secretory Carcinoma of the Parotid: Making the Correct Diagnosis of a Rare Salivary Gland Carcinoma When Molecular Biology Testing Is Not Available

**DOI:** 10.1155/2019/5103496

**Published:** 2019-03-17

**Authors:** Nelson Montalvo, David Galarza, Ligia Redrobán

**Affiliations:** ^1^Facultad de Ciencias Médicas de la Salud y la Vida, Escuela de Medicina, Área de Ciencias Básicas, Cátedra de Patología, Universidad Internacional del Ecuador. Servicio de Patología, Hospital Metropolitano. Av. Mariana de Jesús s/n y Nicolás Arteta. Quito, Ecuador; ^2^Facultad de Ciencias Médicas de la Salud y la Vida, Escuela de Medicina, Docencia y Departamento de Investigación, Universidad Internacional del Ecuador, Ecuador; ^3^Servicio de Patología, Hospital Metropolitano, Ecuador

## Abstract

Secretory carcinoma (SC) is a recently described entity occurring in the salivary glands. Before its description, SC was frequently classified as acinic cell carcinoma (ACC) or adenocarcinoma, not otherwise specified. Its particular histopathological and immunohistochemical characteristics are reminiscent of breast secretory carcinoma. Moreover, it displays a characteristic t(12;15) (p13;q25) translocation that results in the* ETV6-NTRK3* gene fusion. This translocation has not been reported in any other salivary gland carcinoma. Identification of the t(12;15) (p13;q25) translocation is the gold standard for diagnosis, although some cases that do not present this specific translocation have already been reported. In such cases, diagnosis is challenging. In addition, some diagnostic pathology laboratories lack the resources to perform the molecular analysis to diagnose SC. In this scenario, morphology and immunohistochemistry are fundamental. Therefore, we report a case emphasizing the typical morphology of SC and its immunochemical profile to establish a final diagnosis without molecular biology tests. This case aims to demonstrate the importance of recognizing the typical presentation of a rare tumor so that clinicians will be informed or reminded of it and consider this entity among the differential diagnoses, when necessary. Moreover, in low-resource settings where molecular analysis is not available, being familiar enough with the histology of this tumor and using the immunoprofile as a key tool for differential diagnosis would be of great importance in establishing the correct diagnosis. The differential diagnosis includes, above all, acinic cell carcinoma and other salivary neoplasms such as intraductal carcinoma, low-grade mucoepidermoid carcinoma, and adenocarcinoma, not otherwise specified, which is actually a rule-out diagnosis.

## 1. Case Summary

A 27-year-old Hispanic male patient with no relevant clinical history presented with a mass in the right parotid region, adjacent to the angle of the mandible. According to the patient, the mass had slowly but progressively increased in size over a period of 18 months, measuring approximately 1.5 centimeters in its greatest dimension. On physical examination, the mass was painless, firm in consistency, and nonmovable and displayed no changes in the overlying skin. No other masses were found in the face or neck and there was no evidence of facial nerve paralysis. The patient reported no other symptoms, such as xerostomia and sialorrhea.

**Figure 1:**
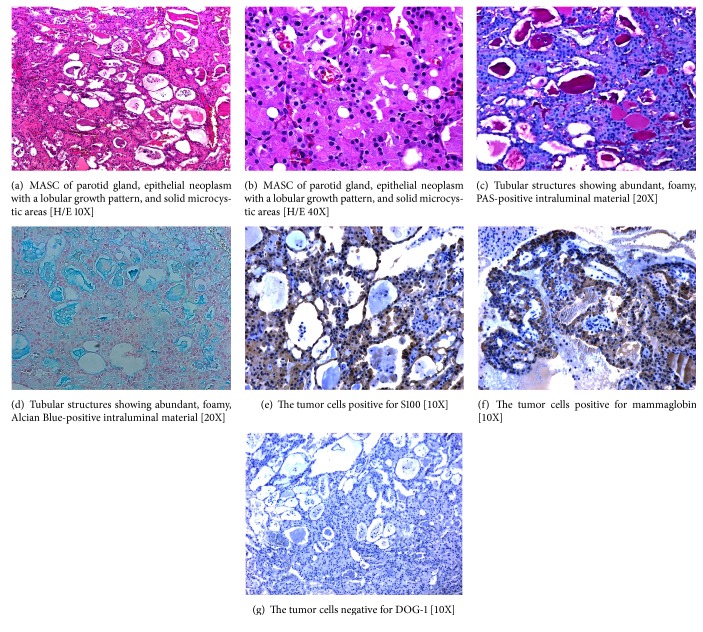
SC of parotid gland, epithelial neoplasm with a lobular growth pattern, and solid microcystic areas (a-b) [H/E 10X and 40X]. Tubular structures showing abundant, foamy, PAS- and Alcian Blue-positive intraluminal material (c-d). The tumor cells positive for S100 and mammaglobin (e-f), but negative for DOG-1 (g).

An incisional biopsy was performed as a first approach to study the lesion. Macroscopic analysis of the biopsy sample was limited because the tissue was fragmented. Microscopic examination revealed an epithelial neoplasm with a lobular growth pattern, dense fibrous connective septa, and solid microcystic areas and tubular structures (Figures 1(a)-1(b)) showing abundant, foamy, PAS- and Alcian blue-positive intraluminal eosinophilic material. The tumor cells were positive for S100, mammaglobin (Figures 1(c)-1(d)), GCDFP15, CD117, CEA-P, and keratin 7 (images not shown), with a cell proliferation index (Ki-67) of 13%. They were negative for DOG-1 (Figure 1(e)), p63, and TTF-1 (images not shown). The histological picture and immunophenotype confirmed the diagnosis of secretory carcinoma of the parotid. A total right parotidectomy was recommended based on this diagnosis.

The advised surgery was not performed soon after the diagnosis, but only six weeks later. Within that period, a local recurrence developed from the original lesion in the same area where the biopsy had been taken. This mass was found during the preoperative check-up. It was painless, firm, and measured 0.5 cm in diameter. Finally, a superficial parotidectomy and a supraomohyoid neck dissection were performed as a definitive treatment. No lymph node showed evidence of tumor cells, and surgical margins were negative. After three days of uneventful postoperative recovery, the patient was discharged from the hospital.

## 2. Discussion

Skálová et al. first described SC in 2010 with the name of mammary analogue secretory carcinoma (MASC) [1]. The most recent WHO publication for the classification of head and neck tumors refers to this entity as secretory carcinoma (SC), since it has been reported to originate at locations other than the salivary glands, such as the skin [2], the lips [3], the thyroid gland [4], the nasal cavity [5], and the lacrimal gland [6]. Although the original name with which this tumor was described is widely spread in the medical literature, we will refer to it as secretory carcinoma, since it is the official designation for this entity [7].

SC shares the histological, immunohistochemical, and genetic characteristics of breast secretory carcinoma, an extremely rare neoplasm that usually affects young patients and generally has an indolent clinical course [1, 8]. SC shows a characteristic t(12;15) (p13;q25) chromosomal translocation that has not been identified in any other salivary gland tumor. This reciprocal translocation leads to the fusion of the* ETV6* gene on chromosome 12 with the* NTRK3* gene on chromosome 15, resulting in the constitutive expression of a chimeric tyrosine kinase protein, which would play a crucial role in the oncogenesis of this tumor [9, 10]. Nonetheless, not all SC cases harbor this specific translocation [11].* ETV6-RET* translocation and* ETV6-MET* fusion have also been reported as molecular alterations associated with SC in some specific cases [12, 13].

SC appears mostly in adults with a mean age of 47 years at diagnosis. As opposed to acinic cell carcinoma, SC has a slightly higher prevalence in men than in women [14]. The parotid gland is the most frequent site of origin, followed by the oral cavity (soft palate, oral mucosa, and lips), the submandibular glands, and the accessory parotid glands [3, 9]. A case of SC in the ethmoidal sinus has been described [15].

Clinically, SC presents as a slowly but progressively growing firm mass that is, as in our case, usually painless or nearly painless (see Table 1). Suzuki et al. reported a SC case whose initial presentation was a cervical adenomegaly at first categorized as a metastatic lymph node of unknown primary with no apparent salivary gland lesion [16].

Histopathologically, SC presents as a well-defined cell proliferation with thin fibrous septa that may or may not show hyalinization and which give the lesion a lobular appearance. The growth pattern is eminently secretory and can be microcystic, tubular, solid, macrocystic, or papillary. A papillary-cystic pattern is common in SC cases, while a solid pattern is predominant in acinic cell carcinomas [17]. The presence of abundant homogeneous eosinophilic secretion positive for mucicarmine and PAS (pre- and postdiastase digestion) is characteristic. Less commonly, a fibrosclerotic stroma may be found with isolated cell islands, mostly located in the center of the lesion. Occasionally, the tumor may show a single large cyst lined with apocrine-like epithelium. Tumor cells have low-grade, round, or oval vesicular nuclei with fine granular chromatin and a small prominent nucleolus. The cytoplasm is usually granulated or vacuolated, with a clear or slightly eosinophilic appearance. Cell atypia is usually mild, and mitotic figures are rare [18–20]. There have been reports of SC cases with high-grade transformation showing a tumoral component consisting of trabeculae-forming anaplastic cells, with frequent perineural invasion, areas of comedonecrosis, conspicuous nuclear polymorphism, and absence of secretory activity. This dedifferentiation phenomenon had already been reported in other types of salivary gland cancer [21].

The immunohistochemical profile of SC consistently shows positivity, usually intense and diffuse, for mammaglobin, S-100 protein, and vimentin [19]. It also tends to variably express pancytokeratin, CK7, CK8, EMA, STAT5a, and GCDFP15 and usually shows negativity for DOG-1 and for basal and myoepithelial cell markers, such as calponin, SMA, CK5/6, and p63. Some cases have been reported with certain peripheral areas that are focally positive for p63 [22, 23].

No single immunohistochemical marker makes the SC diagnosis possible. Several studies have shown that mammaglobin is highly sensitive but lacks sufficient specificity to be an individual diagnostic marker [24, 25]. Expression of DOG-1, a chloride channel selectively present in serous acinar cell and intercalated ductal cell membrane, is useful for improving the specificity of mammaglobin in SC diagnosis. A profile that shows positivity for protein S-100, mammaglobin, vimentin, and adipophilin in combination with DOG-1 negativity is suggestive of SC [24, 26].

The characteristic molecular alteration of SC is the t(12;15) (p13;q25) translocation. This translocation causes the fusion of genes* ETV6* and* NTRK3*, resulting in ligand-independent dimerization of the receptor encoded by the* NTRK3* gene. This activates a signaling pathway that induces cell proliferation leading to neoplastic transformation. Fluorescence in situ hybridization identification (FISH) of this chromosomal alteration is the gold standard for diagnosing SC, since this translocation has not been found in any other salivary gland carcinoma. However, not all SC cases have this typical translocation. Recently, ten cases of SC were described in which the* ETV6*-*RET *gene fusion was identified [12]. In addition, one case reported a* ETV6*-*MET *gene fusion [13]. It is interesting that although not all SC cases show the particular translocation initially described by Skálová, they all show an alteration that involves the* ETV6* gene, a transcription regulator that can fuse with genes other than the* NTRK3* gene. This atypical molecular characteristic may be related to a histological pattern with more infiltrative characteristics and a less favorable clinical prognosis [11, 27].

For diagnosis, the three main characteristics of the original SC description should be considered. First, a histopathological pattern shows morphology suggestive of apocrine secretory epithelium, papillary-cystic or microcystic pattern, abundant PAS-positive eosinophilic secretion, and absence of basophilic zymogen granules in the tumor cell cytoplasm, the latter being a key difference from acinic cell carcinoma. Second, the immunohistochemical profile should include at least mammaglobin, S100 protein, and DOG-1 to guide the diagnosis. Finally, the presence of the t(12;15)* ETV6-NTRK3 *translocation is a finding that unequivocally confirms the diagnosis of SC in the major salivary glands [14]. It is worth noticing that SC can be also found in the sinonasal tract and there the* ETV6-NTRK3* and* ETV6-RET* fusions are found in a subset of sinonasal nonintestinal type adenocarcinoma [28, 29].

In typical cases such as ours, the histopathological study and the immunohistochemical profile are sufficient for diagnosis and do not require molecular confirmation [30]. In cases with uncharacteristic histopathology and nonspecific immunohistochemistry, whose results do not provide sufficient elements for a differential diagnosis, detection of the translocation associated with SC is necessary and relevant.

Differential diagnosis of SC includes acinic cell carcinoma, intraductal carcinoma (low-grade cribriform cystadenocarcinoma), and low-grade mucoepidermoid carcinoma. Acinic cell carcinoma is the most important of these differentials (see Table 2). Chiosea et al. published a review of 81 salivary gland neoplasms originally diagnosed as acinic cell carcinomas [34]. The t(12;15) (p13;q25) translocation was found in 10 of 17 cases showing zymogen granules poor tumors constituted by cells with eosinophilic vacuolated cytoplasm. These cases were reclassified as SC while the* ETV6*-intact cases were retained as acinic cell carcinomas. It is important to point out that a* HTN3-MSANTD3* fusion was recently described in a subset of acinic cell carcinoma, further separating these from SC [31]. Immunohistochemistry also enables differentiating SC from acinic cell carcinoma. The latter is usually negative for protein S100 and mammaglobin and positive for DOG-1. The authors concluded that the occurrence of acinic cell carcinoma outside the parotid is rare and that, in these cases, the SC diagnosis should be considered first.

In general, the clinical course of SC is indolent. The risk of local recurrence and lymph node metastasis is 15% and 20%, respectively. The risk of distant metastasis is around 5% and cases with high-grade transformation have a worse prognosis [35].

Treatment of SC depends on the stage of the disease at diagnosis and on the tumor's histological and molecular characteristics. The treatment of choice for low-grade SC is complete surgical resection. In this scenario, few cases show recurrence. In our case, however, the patient presented recurrence six weeks after initial biopsy, probably due to the presence of residual tumor cells. After the second surgery, no other recurrences were detected during the patient's four-year follow-up.

Locoregional radiation therapy may be considered for large tumors or those that have shown perineural invasion or positive margins. The need for lymph node dissection depends on each case. In cases of SC with high-grade transformation, total resection of the affected gland and adjuvant radiotherapy is recommended. In addition, this type of neoplasm shows a greater propensity to metastasize to the cervical lymph nodes, which would suggest the need for lymph node dissection for optimal management of these patients [36]. In our case, a supraomohyoid neck dissection was performed with no lymph node metastasis.

## 3. Conclusion

Since not all pathology laboratories have the resources to perform the molecular analysis, histological study and immunohistochemistry are key tools for establishing the diagnosis when the clinical presentation, morphology, and immunohistochemical profile of the case typically conform to the SC description, as in our case. Even when a rare case is involved, it is important to keep in mind the typical findings and presentation, since it is probable that a larger number of clinicians will see these cases and thus potentially improve clinical practice. Our case report aims to demonstrate the importance of recognizing the typical presentation of a rare tumor so that a correct diagnosis can be made independently of molecular analysis.

## Figures and Tables

**Table 1:** Clinicopathological characteristics of secretory carcinoma.

*Average age of presentation*	47 years

*Sex*	Equal distribution or a slight male predominance (1.5:1), depending on the series

*Most frequent location*	Parotid glands, without lateral predominance

*Clinical presentation*	Progressively growing painless mass Erythema and ulceration of the overlying skin are not usual Deep plane fixation is variable

*Macroscopic characteristics*	A firm mass with a rubbery consistency to the touch The cut surface is grayish-white and may present small cystic spaces with yellowish secretions

*Microscopic characteristics*	Eosinophilic cell proliferation forming lobules separated by thin fibrous septa and showing microcystic, tubular, papillary, or solid patterns and abundant PAS- and mucicarmine-positive secretion Cells having uniform oval nuclei with loose chromatin and a single prominent central nucleolus

*Immunohistochemistry*	S-100 protein, mammaglobin, and vimentin positive DOG-1, p63, and calponin negative

*Molecular alterations*	Characteristic t(12;15) (p13;q25) translocation with *ETV6-NTRK3 *gene fusion The *ETV6-RET *and* ETV6-MET *gene fusion have also been described in some cases

**Table 2:** Key elements in the differential diagnosis of secretory carcinoma.

	*Secretory carcinoma*	*Acinic cell carcinoma*	*Intraductal carcinoma (low-grade cribriform cystadenocarcinoma)*	*Low-grade mucoepidermoid carcinoma*
*Location*	Parotid gland (75%); present in minor salivary glands, more frequently than ACC, and in the oral cavity	Parotid (90%); very rarely in minor salivary glands	Parotid, most frequently; tongue (posterior region) and minor salivary glands	Parotid (50%) and oral cavity (palate and oral mucosa); very rarely (1-2%) in the submandibular gland Also described in the lacrimal gland, larynx, nose, and paranasal sinuses

*Prevalence by sex*	Slight male predominance	Female predominance	The same for both sexes	The same for both sexes

*Morphological growth patterns*	Predominantly tubular, microcystic, and solid More frequently cystic-papillary than in ACC	Common: solid, follicular, and microcystic Rare: cystic-papillary	Encapsulated and cystic, with cribriform and papillary patterns	Heterogeneous pattern: solid and cystic with hydropic degeneration and metaplastic changes

*Cell morphology*	Epithelial, without acinar differentiation	Acinar and basophilic	Monotonous, with ductal, cuboidal, and apocrine characteristics	Morphologically bland epidermoid, mucinous, and intermediate cells that are oncocytic, clear, or columnar/polygonal

*Cytoplasm*	Eosinophilic, granular, or vacuolated; no zymogen granules	PAS positive zymogen granules*∗*	Eosinophilic, very infrequently with iron pigment	Abundant, clear (mucicarmine, Alcian blue, and PAS-diastase positive), eosinophilic, and foamy

*Nuclei*	Round or oval	Monomorphic	Clear vesicular nuclei with the appearance of frosted glass that overlap one another	Small hyperchromatic nuclei

*Immunohistochemistry*	S-100 protein and mammaglobin positive Usually positive for STAT5a and DOG-1; p63 negative	Mammaglobin and p63 negative S-100 protein usually negative DOG-1 intensely positive with apical pattern	S-100 protein, vimentin, and mammaglobin positive; p63- and calponin-positive myoepithelial cells	Positive p63 staining in epidermoid foci and usually S100 and mammaglobin negative

*Molecular Alteration*	t(12;15) *ETV6-NTRK3,* 80% of cases Rearrangements in the *ETV6* gene, 99% of cases (*ETV6-RET* and *ETV6-MET* fusion reported)	*HTN3-MSANTD3* fusion described in a subset of cases [31]	*NCOA4-RET* and *TRIM27-RET* fusion genes [32, 33]	t(11;19)* CRTC1-MAML2* t(11;15) *CRTC3-MAML2*

*∗* There is a type of acinic cell carcinoma whose cells contain few zymogen granules. In these cases, the differential diagnosis of SC relies mainly on molecular analysis.
